# Evidence-Based Policies on Migration and Global Health Are Essential to Maintain the Health of Those Inside and Outside the United States

**DOI:** 10.4269/ajtmh.961ed

**Published:** 2017-01-11

**Authors:** Philip J. Rosenthal, Daniel G. Bausch, Stephen Higgs, N. Regina Rabinovich, David R. Hill, Christopher V. Plowe, Karen A. Goraleski, Patricia F. Walker

**Affiliations:** 1 Editor-in-Chief.; 2 Scientific Program Chair.; 3 Immediate Past President.; 4 President-elect.; 5 Secretary-Treasurer.; 6 Past President.; 7 Executive Director.; 8 President.

Human migration is at an all-time high, with one in every 122 humans now either a refugee, internally displaced, or seeking asylum (unhcr.org/news/latest/2015/6/558193896/worldwide-displacement-hits-all-time-high-war-persecution-increase.html). This global humanitarian crisis has spurred vigorous local, national, and international debates regarding the risk of infectious diseases that migrants pose to the countries and citizens who offer them shelter. In these debates, some have advocated tightened control and oversight of immigration, acceptance of refugees, and international travel. In this context, it is essential to maintain established U.S. policy toward human migration and global health that is evidence based and upholds the value of compassion, as well as key principles in international human rights law.

Although travel restrictions may be warranted under extreme circumstances of epidemics and pandemics, as spelled out by the World Health Organization International Health Regulations (who.int/topics/international_health_regulations/en/), experience informs us that limiting migration and the flow of refugees will, by itself, have little impact on the control of infectious diseases. Migrants represent only a small fraction of international travelers entering the United States. Thus, preventing their entry will not have a major impact on the influx of infections. International travel, once considered exotic, is now commonplace. Over 70 million U.S. citizens travel abroad yearly for commerce, trade, tourism, teaching, faith-based missions and other public service efforts, and military deployment. In contrast, about 1 million legal immigrants and 70,000–90,000 refugees enter the United States yearly.

Unlike U.S. citizens, who travel freely without health screenings or restrictions, legal migrants and refugees who enter the country must meet entry criteria that include thorough screening and treatment of infectious diseases. American travelers returning to the United States have proven to be highly efficient in spreading some infectious diseases. For example, of the almost 4,500 recognized cases of Zika virus infection to date in the United States, over 95% were travel related (cdc.gov/zika/geo/united-states.html). Furthermore, some “tropical” infections we may fear from migrants are already endemic, albeit uncommon, in parts of the United States, including viral infections, tuberculosis, Chagas disease, leishmaniasis, and soil-transmitted helminth infections. Restrictions on travel and population movement cannot keep these diseases out of our country. They are already here.

In addition to maintaining a humane and evidence-based U.S. policy on migration, we must continue to engage and invest in programs that improve the health of vulnerable populations worldwide. Reaching out to enhance the well-being of those whose lives has been torn apart by war and oppression should be a fundamental and perhaps defining American principle. Programs for populations in need around the world should not be considered antiquated historical notions or reflective of an outmoded inscription on the Statue of Liberty. Indeed, U.S. government leaders from both sides of the political aisle have championed programs aimed at global health, including the creation of the Peace Corps by President John F. Kennedy in 1961, and the President's Emergency Plan for AIDS Relief and President's Malaria Initiative by President George W. Bush in 2003 and 2005, respectively. Additional efforts have been shared with other developed countries, notably the Global Fund to Fight AIDS, Tuberculosis, and Malaria. These well-managed programs have offered enormous benefits to the citizens of the developing world, but they have also directly benefitted the United States, by helping to control some of the most important infectious diseases that threaten all of us, by building diplomatic bridges of good will with populations around the world, and by exemplifying the best of American values of kindness and compassion.

Progress in diagnosing, managing, and preventing tropical infectious diseases will be facilitated by biomedical research in the developing world, where greater prevalence of many infections allows adequate sample size for studies that would not be possible through research conducted only in the United States. New drugs, vaccines, and control measures that result from these research efforts will be available for the benefit of all, including those in the United States. Maintaining this research will require continued funding for U.S.-based agencies and programs that support global health research and service, including the National Institutes of Health, Centers for Disease Control and Prevention, U.S. Agency for International Development, and many Department of Defense programs.

The American Society of Tropical Medicine and Hygiene, the largest international organization of experts dedicated to reducing the worldwide burden of tropical infectious disease and improving global health, deeply understands that vigilance in the control of global disease is in the best interest of all. To that end, we will continue to work with the U.S. Congress, the White House, and global health stakeholders in support of evidence-based policies and programs to protect the health of people both inside and outside the United States. Maintenance of evidence-based policies concerning migration and strong investment in global infectious disease research and programs are the right thing to do, and they are the smart thing to do.

